# Birch Wood Surface Characterization after Treatment with Modified Phenol-Formaldehyde Oligomers

**DOI:** 10.3390/polym14040671

**Published:** 2022-02-10

**Authors:** Juris Grinins, Mairis Iesalnieks, Vladimirs Biziks, Ineta Gritane, Guntis Sosins

**Affiliations:** 1 Latvian State Institute of Wood Chemistry, 27 Dzerbenes Str., LV-1006 Riga, Latvia; mairis225@inbox.lv (M.I.); gritaneineta2@inbox.lv (I.G.); guntissosins@gmail.com (G.S.); 2 Institute of Wood Biology and Wood Products, Georg-August University Göettingen, Büsgenweg 4, 37077 Göttingen, Germany; Vladimirs.Biziks@surfactor.com

**Keywords:** birch wood, color stability, fatty acid chlorides, hydrophobicity, phenol-formaldehyde resin

## Abstract

Phenol-formaldehyde (PF) resins with well-established molecular sizes are promising treatment agents for wood bulk protection. However, due to the presence of hydroxyl groups on the periphery, the PF oligomers tend to absorb the water, which can lead to water penetration into the wood. To overcome this drawback different PF pre-polymers have been chemically modified with different long-chain fatty acid chlorides (FAC) via esterification. To obtain the modified PF (M-PF) resins, the PF pre-polymers with average molecular weight (M_w_) from 266 to 884 g/mol were esterified with decanoyl, lauroyl, myristoyl, palmitoyl, and stearoyl chloride in pyridine as the reaction medium. Silver birch (*Betula pendula*) wood specimens (15 × 70 × 150 mm^3^) were coated with M-PF pre-polymer 5% (w/w) solutions in tetrahydrofuran (THF), and hydrophobic properties of treated birch wood specimens were evaluated using surface contact angle (CA) measurements of water droplets. For all M-PF resin-treated specimens, CA was almost 2–2.5 times higher than for untreated wood (45°) and it remained 80–125° after 60 s. The aging properties of M-PF resin-coated birch wood were analyzed using artificial weathering with ultraviolet (UV) light and combination of both UV and water spray. Results clearly confirm, that the hydrophobic properties of M-PF-treated wood has short-term character and will gradually disappear during long-term application in outdoor conditions.

## 1. Introduction

The effect of UV irradiation and of the repeated wetting and re-drying of wood surfaces can be reduced to some extent by four approaches:

1. Using coatings. Generally, wood surfaces are protected using opaque coatings with pigments. Although these coatings considerably decelerate deterioration of the wood substrate, the natural appearance and texture of the wood are hidden (lost) under the pigments. To overcome this drawback, translucent coatings are used. For clear coatings that transmit solar radiation, the wood beneath the coating degrades, resulting in the failure of the coating at the wood-finish interface. The performance of clear coatings on wood can be improved by employing two different strategies [1]. The first one focuses on modifying coating formulations to adapt the properties of the coating systems and thus meet the requirements of the wood substrate. For this reason, coating flexibility has been in the focus of much research. Several clear and semi-transparent coatings on wood have been tested and it was found that formulations with a lower glass transition temperature (Tg) have lower rates of crack formation and thus longer service lives than formulations with a higher Tg [2,3,4]. The second strategy is to introduce different UV absorbers and radical scavengers (hindered amine or phenolic light stabilizers) into formulations to reduce the degradation of the coating and the underlying wood substrate. Although this strategy has been used for clear coatings, the wood beneath the coating still degrades, albeit at a reduced rate, resulting in coating failure [5]. However, these coatings do not improve the intrinsic wood properties. Consequently, once cracks appear in the coating, water can easily penetrate the substrate. Therefore, the dimensional stability and biological durability of wood are still important factors [6].

2. Chemical treatment of wood. A number of studies have focused on improving both the weathering resistance of wood through chemical modification and the color stability during accelerated or outside weathering. Compared with similarly exposed untreated wood, acetylated [7,8], glutaraldehyde [9], DMDHEU-, and melamine- [10] treated wood exposed to accelerated or outside weathering develops fewer cracks because of its improved dimensional stability. Surface discoloration and crack formation during longer exposure times are reduced, whereas in the case of thermal treatment, the rate of graying and crack development is the same or even faster than that of untreated wood [11,12]. Mantanis and Lykidis [13] have evaluated the weathering performance of furfurylated wood decks of radiata pine, maple, and yellow pine using the untreated tropical hardwood Ipe (Tabebuia) as a reference. The Ipe wood surface exhibited cracks and discoloration, and the Ipe deck was more distorted than the treated ones. The phenol formaldehyde (PF)-treated boards remained darker, ranging from light brown to dark brown. This change in optical appearance depends on both the resin type and the average molecular size of the PF resin oligomers used for treatment. The surface of PF-treated wood without a coating has improved resistance against photodegradation compared to the surface of N-methylol melamine (NMM)-treated wood because PF inhibits lignin degradation [14].

3. Coating on chemically treated wood. The performance of coatings on heat-treated wood can vary according to the wood species used rather than the thermal modification process [4,15]. The reflectance of a UV-stabilized acrylic clear coat on acetylated Scots pine was virtually unchanged after 1728 h of accelerated weathering [16]. It was concluded that acetylation can prevent the discoloration or failure of a fully transparent coating after severe artificial weathering. Evans et al. [3] investigated the influence of acrylic coatings on NMM- and PF-treated wood substrates and observed that the PF resin in particular has good potential to improve the weathering performance and prolong the service life of wood. However, the method by which a wood substrate is pretreated prior the application of coatings on the surface is important. The performance of three different clear coatings (polyurethane, silicone, and polyacrylate) on PF-treated wood after three different pretreatment methods: immersion, brushing, and impregnation was tested [17,18]. Coatings performed better on impregnated wood than on wood immersed in or brushed with resin.

4. By producing superhydrophobic surfaces. A superhydrophobic treatment of wood has the potential to improve water and dust repellency, increase dimensional stability and extend service life. Water droplets on a superhydrophobic surface remain nearly spherical and easily roll off, removing pollutants and engendering self-cleaning properties. Lu et al. [19] have tested the weathering performance in terms of color stability and wettability of a poplar wood surface treated with cerium oxide and covered with a layer of octadecyltrichlorosilane (OTS). The resulting coating exhibited the properties of a superhydrophobic wood surface as contact angle (CA) was approximately 152° and, compared to the control, exhibited an effective reduction in UV irradiation-induced color changes, indicating excellent UV resistance. Although the outside-facing part of the wood material is protected using this approach, the internal structure remains untreated; hence, dimensional changes in the wood may occur during water sorption. In addition, most reported superhydrophobic wood surfaces have some disadvantages, e.g., a top coating has limited mechanical stability, glue-ability and durability, and additionally, the processes are time consuming and require a tedious preparation process. A novel and simple method to prepare highly hydrophobic (CA of 146°) wood with its dimensional stability improved by 30% was used [20]. The long alkyl chains were chemically bonded (grafted) onto the cell walls via reaction between the hydroxyl groups in the wood and the isocyanate groups of octadecyl isocyanate (OTI), thus forming a stable bulk hydrophobic structure. These excellent hydrophobic properties were attained throughout the entire wood sample, i.e., not only on the surface but also in the core of OTI-treated wood, where the small dimension (10 × 10 × 10 mm^3^) specimens were used.

Among all the listed methods, the most promising weathering performance of a wood surface was obtained using a combination of a flexible coating and chemically modified wood, specifically, PF-treated wood (bulk treatment) coated with a flexible water-based acrylic varnish. Another promising approach is grafting (chemically bonded) hydrophobic long alkyl chain compounds, e.g., OTS or OTI, onto the wood bulk. However, in the first approach for achieving high weathering performance of wood, two separate treatment steps must be implemented: wood treatment (impregnation, drying, and curing) and then coating or a subsequent hydrophobization step with waxes, paraffin etc. In the second approach, the surface properties (shell) of wood are more substantially altered than the core properties. Specifically, the wood shell exhibits superhydrophobic properties, whereas the core remains unchanged.

PF resins with well-established molecular sizes are promising treatment agents for wood bulk protection. However, the presence of hydroxyl groups on the periphery of PF oligomers can be assumed to be a disadvantage. PF-treated wood tends to absorb and take up water and water vapor, which can lead to water penetration into the wood, reduced cracked formations and, to some extent, swelling. Hygroscopic properties of six polymer films obtained from commercial wood adhesives such as phenol-resorcinol-formaldehyde (PRF), melamine-urea-formaldehyde resin (MUF), fish glue, polyvinyl acetate and two polyurethanes using dynamic vapor sorption (DVS) analysis have been evaluated [21]. Both polymer films made of thermosetting resins had strong water absorption and achieved high weight gains, with PRF accumulated 18% water, while MUF absorbed 22%. Absorption of water in polymer matrix has the potential of causing undesirable effects. Even a small amount of water uptake in polymer may cause additional internal stresses and thereof influence the mechanical performance, or fungal attack may occur in case polymer moisture reaches higher levels. This weakness of PRF resin can be overcome to some extent by chemical modification of PRF resin monomer.

The repeated wetting and re-drying of wood surfaces and negative effect of UV irradiation can be reduced to some extent by using the surface coating, chemical treatment, or coating chemically modified wood. These disadvantages can be overcome by implementing another approach based on wood treatment with modified phenol-formaldehyde (M-PF) resin pre-polymers. Such treatment might result in superhydrophobic surface of wood and has the potential to improve water and dust repellency, increase dimensional and color stability, and extend service life.

This study investigated a new approach of birch wood surface treatment with different molecular weight (M_w_) PF resins modified with different chain length fatty acid chlorides (FAC) to understand the effect on surface hydrophobicity, weathering performance, and color stability in accelerated laboratory conditions. Theoretically, the photostability and surface hydrophobicity of the wood material treated with M-PF resins should be improved. Criteria for birch wood surface treatment with M-PF resins was based on water droplet surface CA with high values, therefore indicating hydrophobization effect.

## 2. Materials and Methods

### 2.1. PF Resin Pre-Polymer Synthesis

For the synthesis of PF resins, phenol was hydroxymethylated under alkaline reaction conditions, whereby the molar ratios of formaldehyde/phenol/sodium hydroxide were 1,5-1-0.1, 1,5-1-0.2, 1,8-1-0.15, 2-1-0.2, and 2-1-0.4. During the synthesis of each resin, a measured amount of phenol (99%, Acros Organics) and sodium hydroxide (98.5%, Acros Organics) water solution (50% w/w) was weighed out in a 4-neck laboratory reactor (0.5 L) equipped with a thermometer, dropping funnel, reflux condenser, and Teflon stirrer. Ethanol was also added in order to maintain a homogeneous reaction. The 4-neck reactor was submerged in a thermostatic water-bath. As soon as the temperature in the flask reached the necessary synthesis temperature, the aqueous formaldehyde (37% in water, stabilized with 5–15% methanol, Acros organics) solution was added slowly via a drip over a 25–30 min period. The reaction temperature (65, 75, 85 °C) for each formaldehyde/phenol/sodium hydroxide molar ratio was kept constant during the entire reaction period (2 and 4 h). The resol synthesis was ended by cooling the reactor with cold running water and allowing the resol to cool down to 20 ± 3 °C. Totally 15 different PF resins were synthesized and tested for modification reactions with long chain FAC. Based on surface wettability after water droplet surface contact angle (CA) measurements, nine PF resins were selected for further tests. Synthesis parameters of PF resins selected for modification with FAC are listed in Table 1.

#### PF Resin Characterization

The dynamic viscosity of liquid PF resins was determined by a Fungilab Viscolead Adv (Fungilab S.A., Barcelona, Spain) meter with a suitable spindle. The non-volatiles content (solid content) was determined according to DIN EN ISO 3251:2019 [22]. The pH value was determined using a digital pH meter (GPH 114 Greisinger, Regenstauf, Germany) by inserting the pH meter electrode into the PF resins. The pH meter was calibrated with buffer solutions at pH 4.0 and 10.0 prior pH measurements. Free formaldehyde content was determined by the hydroxylamine hydrochloride method according to DIN EN ISO 9397:1997 [23].

For gel permeation chromatography (GPC) analysis, a 1260 Infinity system (degasser, isocratic pump, automatic liquid sampler, heatable column compartment, RID, MWD @ 280 nm), Agilent (Santa Clara, CA, USA) was used, where: column: 3 x PLgel 5µ (50 Å, 100 Å, 1000 Å), 7.5 × 300 mm^2^; solvent: tetrahydrofuran (THF); flow rate: 0.6 mL/min; flow rate marker: acetone; calibration: polystyrene standard.

Approximately 40 mg of resin was dissolved in 5 mL of THF. If the resin did not completely dissolve, it was sonicated with H_2_SO_4_, which was slowly added (5% in methanol) until neutral. If the resin was dissolved, but precipitate from additives (such as salts) remained, the mixture was filtered with a syringe filter.

### 2.2. Modification of PF Pre-Polymers

For the next step in the synthesis of hydrophobically modified PF (M-PF) resin, the presence of water was not desirable. Therefore, the remaining formaldehyde and water were evacuated by vacuum-rotary distillation at 40 °C for 90 min and then further removed using a vacuum drier at room temperature for 12 h at 150 mbar vacuum.

To prepare hydrophobically the M-PF resins, 2,4,6-trimethylolphenol was esterified with decanoyl chloride C_10_ (98 + %, Acros Organics), lauroyl chloride C_12_ (98%, Acros Organics), myristoyl chloride C_14_ (97%, Acros Organics), palmitoyl chloride C_16_ (98%, Acros Organics), and stearoyl chloride C_18_ (>97%, Tokyo Chemical Industry Co. Ltd., Tokyo, Japan) in pyridine as the reaction medium at 50 or 60 °C for 2 and 3 h under a positive pressure of nitrogen gas. Reactions were conducted in the same four-neck flask equipped with a water-cooled condenser, a thermometer, a Teflon stirrer, and a dropping funnel, which was used to add long-chain fatty acid chlorides. A sample (~0.1 g) was removed from the flask after 1 h and was mixed together with ethanol to ascertain that the hydrophobization had occurred. Unreacted FAC can react with ethanol to form ethanol-soluble ester, whereas the esterified PF pre-polymer (monomer-oligomer mixture) is precipitated. The content in the flask was cooled down on ice after 2 or 3 h, and ethanol was added. The M-PF resin pre-polymer then underwent the purification steps: 4 washing-precipitation cycles in ethanol and subsequent usage of a centrifuge (7000 rpm for 10 min). After purification and vacuum drying for 1 day, reaction yield of solid M-PF resin pre-polymer was obtained. The synthesis parameters for PF resin reaction with FAC are listed in Table 2. Nine different PF pre-polymers, two synthesis temperatures (50 and 60 °C), two synthesis durations (2 and 3 h), two FAC/PF resin pre-polymer (mol/g) ratios and the reaction yields were evaluated. None of the PF resins modified with decanoyl chloride C_10_ showed a sufficient hydrophobicity effect on the birch wood surface and only FAC with chain length C_12_–C_18_ was used for further tests.

**Figure 1:**
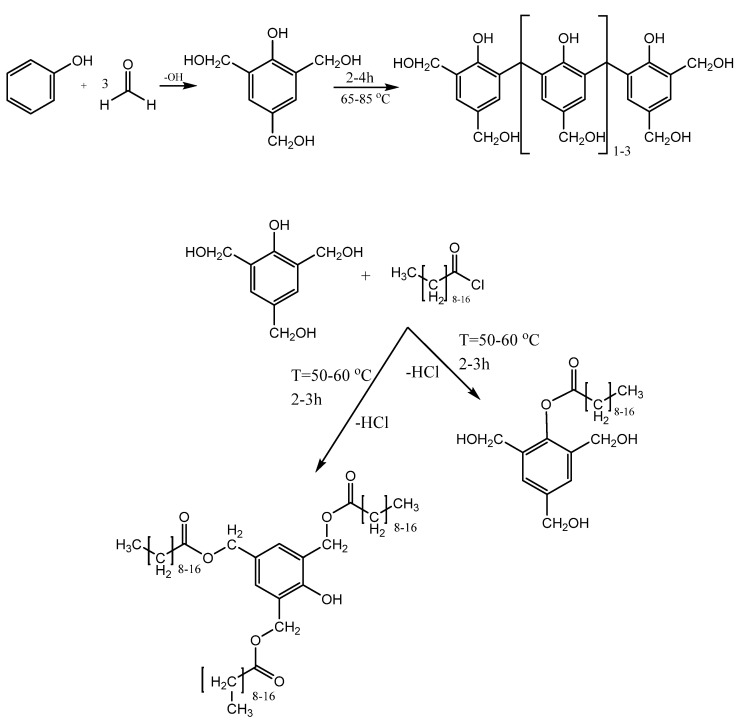
Theoretical reaction mechanisms for M-PF resin synthesis.

Theoretical reaction mechanism of esterified PF resin synthesis is shown in Figure 1. Theoretically, all four hydroxyl groups in 2,4,6-trimethylphenol can be substituted. However, this outcome is not desirable because the thermal reactivity of the modified monomer will be considerably decreased due to the degree of substitution. The presence of primary –OH groups such as hydroxymethyl groups is important for the last step in wood treatment: curing, when the auto-condensation reaction occurs within the resin.

The easiest would be the hydrophobization of all monomers with a hydroxymethylation degree of 1. In this case, the hydrophobization will occur via the primary –OH groups, but due to high degree of hydroxymethylation, the monomer will still have at least two active hydroxymethyl (-CH_2_-OH) groups that can auto-condense during heating. The second and more laborious method is to functionalize the monomer via the secondary –OH groups.

Hydroxymethylated phenol used as the carrier molecule contains primary and secondary hydroxyl groups. These groups could begin to compete during the esterification reaction (hydrophobization) between the hydroxymethylated phenols or other hydroxymethylated phenolic derivatives and long-chain FAC. Primary hydroxymethyl groups (-CH_2_-OH) on a phenol ring are relatively reactive compared to secondary hydroxyl groups (the phenolic -OH group on the benzene ring). Although the hydrophobization of the primary (-OH) groups is dominant, this reaction pathway is undesirable.

#### M-PF Resin Characterization

M-PF structure was analyzed by Fourier transform infrared (FTIR) spectrometry data, which were obtained with a Thermo Scientific Nicolet iS50 spectrometer (Thermo Fisher Scientific, Waltham, MA, USA) at a resolution of 4 cm^−1^, 32 scans. The FTIR data were collected using attenuated total reflectance technique with ZnSe and diamond crystals.

### 2.3. Treatment of Wood Specimens

Silver birch (*Betula pendula*) wood blocks (15 × 70 × 150 mm^3^ Radial × Tangential × Longitudinal direction) were coated with M-PF pre-polymer 5% (w/w) solutions. Because of their hydrophobic nature, the M-PF pre-polymers were dissolved in tetrahydrofuran (THF). Specimens were coated with obtained emulsions using brush treatment with pre-drying in air for 15–20 min and subsequently second layer was applied on the surface. The coated specimens were pre-dried in air and then oven dried with moderate air circulation and air exchange for 24 h using incrementally rising temperature intervals from 60–103 °C and cured at 140 °C for 1 h. Before being used for further tests the specimens were placed in conditioning room at 65 ± 3% relative humidity and 20 ± 2 °C temperature to reach 5–6% relative moisture content.

### 2.4. Surface Contact Angle (CA) Measurements

Contact angle (CA) was determined by the static sessile drop method: For each treatment, the CAs of two wood specimens with dimensions of 15 × 70 × 150 mm^3^ were determined with goniometer OCA20 (Dataphysics, Filderstadt, Germany), equipped with a video camera. Surfaces were not sanded or sliced before CA measurements. The probe liquid was distilled water; each droplet had a volume of 10 μL, and data were recorded for each droplet for 60 s at a fixed interval of 1 s. For each wood specimen, 10 measurements were performed and the CA values were displayed during 1–60 s test period.

### 2.5. Artificial Weathering Tests

Artificial weathering tests were performed in a QUV accelerated weathering tester, (Q-Lab Europe, Ltd., Farnworth Bolton, England) equipped with UVA-340 type fluorescent lamps. Two birch wood specimens (15 × 70 × 150 mm^3^) were used for each M-PF resin treatment. The grain direction of specimens was selected to be parallel to M-PF resin-covered surface (150 × 70 mm^2^). The lamps provided a good simulation of sunlight in the short wavelength region; from 295 nm to 365 nm, with a peak emission at 340 nm. The UV radiation flux density at 340 nm was 0.89 W/m^2^ and the chamber temperature throughout the test was kept constant at 60 °C. The intensity of the full UV spectrum’s (290–400 nm) irradiation was 21.5 W/m^2^. In the study, two different artificial weathering tests were carried out. The first test involved only UV irradiation. This test was regularly suspended to measure the change in color of the specimens. The total duration of the test was 360 h. The second artificial weathering test involved both UV irradiation and water spray. The test involved the following steps; 2.5 h of UV radiation at the same conditions as described earlier, followed by 30 min of water spray. In total, 60 cycles were preformed to reach an exposure time of 180 h from which 150 h accounted for UV irradiation. Color measurements after both weathering methods was performed.

### 2.6. Surface Color Measurements

Color of the specimens was measured with a CM-2500 d spectrophotometer (Konica Minolta, NJ, USA) and expressed according to the CIELAB three-dimensional color system. On each of the specimen, five locations were randomly chosen and marked. For the marked locations, the color was measured before and after the weathering tests as well as during the test after 2, 4, 8, 16, 24, 48, 72, 120, 192, 264, and 360 h. The color was measured to evaluate the discoloration caused by weathering. The total color change Δ*Eab* was calculated according to the equation (1) below. L* is the lightness parameter, a* is the chromaticity parameter which represents red-green coordinates, and b* is the chromaticity parameter which represents yellow-blue coordinates.

(1) $$\Delta Eab=\sqrt{{\left(L_{x}^{*}-L_{o}^{*}\right)}^{2}+{\left(a_{x}^{*}-a_{o}^{*}\right)}^{2}+{\left(b_{x}^{*}-b_{o}^{*}\right)}^{2}}$$

where:

L*_o_, a*_o_, b*_o_ is the value on coordinate axis for the specific parameter at the beginning;

L*_x_, a*_x_, b*_x_ is the value on coordinate axis for the specific parameter after weathering.

## 3. Results

### 3.1. Surface Contact Angle (CA)

**Figure 2:**
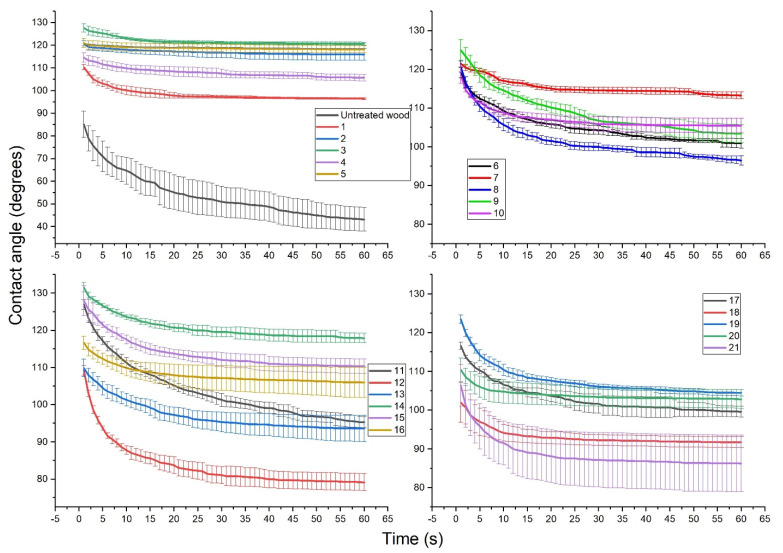
Water droplet surface contact angle (CA) as a function of time for untreated and modified PF resin-covered birch wood.

The contact angle (CA) of a drop (10 µL) of deionized water on the radial surface of birch wood was recorded, and the changes in contact angle over 60 s were assessed (Figure 2). For all M-PF resin-treated specimens, CA was larger than for untreated wood (45°), and it remained between 80 and 125° after 60 s. Additionally, all M-PF treated specimens had decreased slope in CA reduction compared to untreated during the first seconds of measurement. CA measurements in the dynamic mode showed that huge deviation in hydrophobicity was influenced by the length of FAC. The highest CA values (115–125°) after 60 s were observed for specimens No. 2, 3, 5, 7, 14, and 15 which were treated with palmitoyl chloride-C_16_ and stearoyl chloride-C_18_ M-PF pre-polymers. Depending on FAC chain length used for PF pre-polymer modification, the CA values after 60 s were 90–108° for C_12_, 85–100° for C_14_, 100–120° for C_16_, and 80–125° for C_18_. It seems that using a longer chain, FAC resulted in higher maximum surface hydrophobicity. PF pre-polymers of A, B, and F (M_w_ = 266, 515 and 292 g/mol) which were modified with a longer FAC (palmitoyl chloride-C_16_ or stearoyl chloride-C_18_) were the most suitable for achieving CA of 115–125°.

Our previous studies showed that for plywood made of PF resin (M_w_ = 292, 528, 884 g/mol) impregnated birch veneers, CA values between 80 and 95° after 30 s can be obtained and plywood surface was not classified as very hydrophobic [24]. Thus, modification of PF resin hydroxymethyl groups with FAC significantly improves the resin performance in terms of surface hydrophobicity. Similar to our findings, [25] and [26] used long-chain aliphatic compounds to turn the wood surface from hydrophilic to hydrophobic. For example, [25] used the octadecyltrichlorosilane (OTS) epoxy/silica coating on larch (*Larix gmelinii*) wood and obtained CA between 125 and 155°, low water uptake after immersion, and promising mechanical stability in sand collision test. Whereas, [26] coated the spruce wood with a mixture of tung oil and natural beeswax, followed by the deposition of micronized sodium chloride particles. Using water-soluble micronized saltwater, CA above 160 ° was obtained.

### 3.2. M-PF Resin FTIR Spectra Analysis

**Figure 3:**
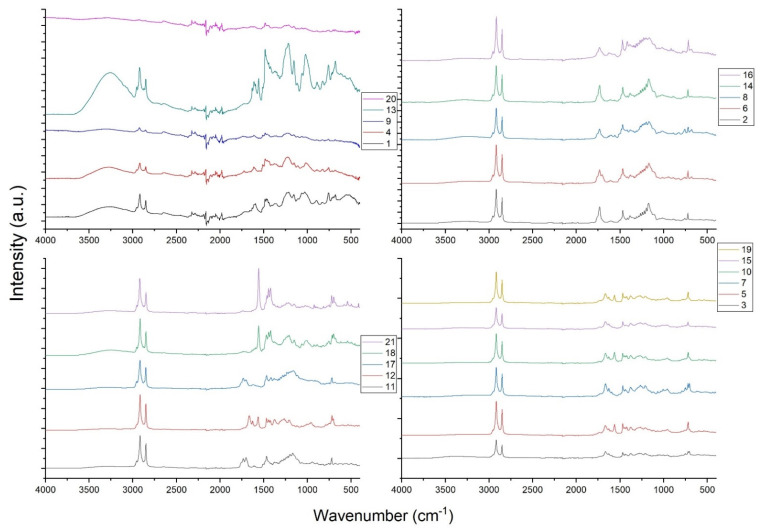
Fourier transform infrared (FTIR) spectra of M-PF resins.

The FTIR spectra of M-PF resins are presented in Figure 3. FTIR spectra were grouped by spectral similarity. Specimens 1, 4, 9, 13, and 20 had significantly different spectra from others and high-quality spectrum could not be obtained for specimens 9 and 20. Specimens 2, 6, 8, 14, and 16 were modified with palmitoyl chloride (C_16_) so their spectra are very similar. Specimens 3, 5, 7, 10, 15, and 19 were similar as modified with stearoyl chloride (C_18_), except 19 which was modified with palmitoyl chloride (C_16_).

Asymmetrical and symmetrical stretching of -CH_2_ and-CH_3_ of long chain fatty acid appeared at 2915 cm^−1^ and 2871 cm^−1^, respectively. Carbonyl of ester showed absorption band at 1667 cm^−1^, strong peak C=O stretching vibrations at 1732 cm^−1^, and >C=O ester stretching at 1265 cm^−1^. Aromatic –OH stretching appeared at 718 cm^−1^ indicating that secondary –OH groups in 2,4,6-trimethylphenol were not substituted with aliphatic chains in reactions with FAC.

Peak at 702 cm^−1^ can be attributed to C-C skeletal vibration in fatty acids. Peak at 1172 cm^−1^ corresponds to the C-O-C stretching vibrations associated with the long-chain esters. Peaks at 1467 and 1443 cm^−1^ can be associated with R-CH_3_ and C-H bond asymmetric deformation vibrations and -CH_2_- C-H scissor vibrations, respectively. Peaks at 1561 and 1422 cm^−1^ correspond to pyridine C=C and C=N in plane vibrations meaning that there are traces of pyridine left after the washing with ethanol.

The other characteristic absorptions for asymmetric and symmetrical stretching for -CH_2_, -CH_3_ of fatty acid component of the polymer were observed at 2953 cm^−1^ and 2849 cm^−1^ respectively. Similar FTIR analysis was done with polyurethane wood-finished coatings prepared from vegetable oil-based fatty acid and dimer fatty acid [27]. Transparent wood coatings were obtained with good mechanical properties and authors expected that such coating may increase the shelf-life of wood substrate. However, the surface color stability in intensive UV light or outdoor conditions was not studied.

### 3.3. Surface Color and Hydrophobicity after UV Test

**Figure 4:**
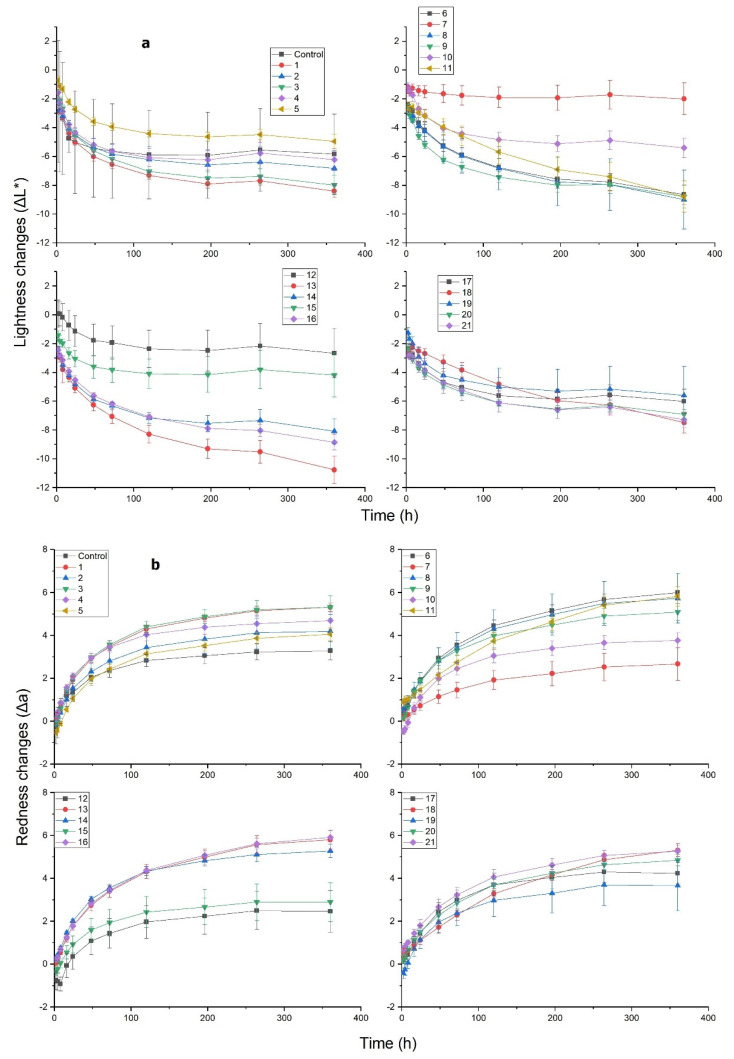

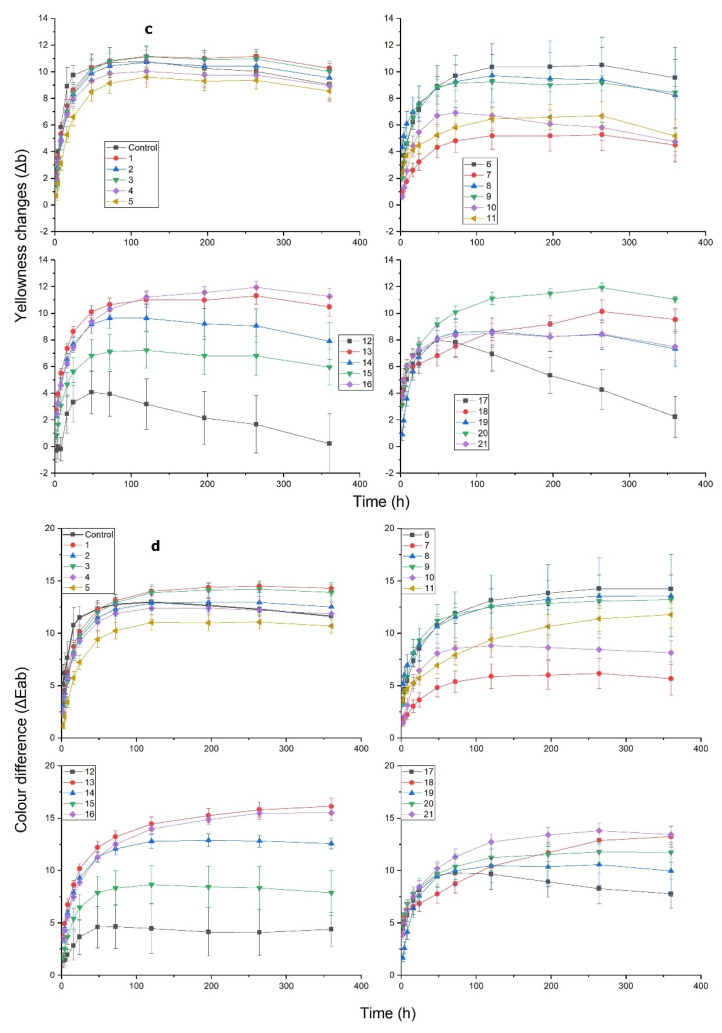
Changes in color of untreated (Control) birch wood and M-PF resin-coated wood after exposure to UV light during 360 h in QUV camera: (**a**) Changes in the CIE parameter ΔL* (lightness); (**b**) changes in the CIE parameter Δa (red-green); (**c**) changes in the CIE parameter Δb (yellow-blue); (**d**) color difference parameter Δ*Eab*.

M-PF resins are transparent and birch wood coated with them retains its natural texture and the surface color changes only slightly after their application. In real outdoor conditions, wood surface color stability is also an important parameter. Wood products with improved color stability naturally have an advantage over competitors. Transparent wood coatings are favorable in terms of maintaining wood natural color and texture. In order to evaluate how UV radiation affects the M-PF resin-coated birch wood developed in this study, color parameters were evaluated at different exposure intervals. The results presented in Figure 4 show color parameters of M-PF-treated and untreated birch wood after UV weathering of 360 h. All M-PF-treated birch wood specimens retained natural texture and with naked eye color difference after treatment is small. Both the untreated controls and the M-PF resin-treated wood specimens became darker (Figure 4a) after UV weathering (decreasing ∆L*). For untreated birch wood, ∆L*decreased by 5 units while for M-PF it was 1–10 units.

Untreated and M-PF resin-treated wood became redder (Figure 4b) during UV weathering (increasing Δa). The change in red color parameter was 3 units for untreated wood and 2–6 units for M-PF resin-treated wood.

The yellowness (Δb) parameter increased similarly to between 6 and 11 units after 360 h for untreated and most of M-PF specimens, except for specimens 12 and 17 (Figure 4c).

The total color change (Figure 4d: Δ*Eab*) for all M-PF treated wood specimens was between 5 and 15 units after 360 h of exposure. Untreated birch wood Δ*Eab* was 12 units at the end of test and only specimens coated with M-PF resins 5, 7, 10, 12, 15, and 17 showed lower values with 4–11 units. PF resins B, D, E, F, and F (Mw = 515, 566, 273, 292 and 884 g/mol) modified with stearoyl chloride C_18_ and myristoyl chloride C_14_ (for specimen 17) were used to obtain these compounds. There was no clear correlation between used PF resins and their molecular weight, but longer chain length for FAC seems to ensure better color stability in UV weathering test.

Spruce wood coated with surfactant-free emulsions based on tung oil, linseed oil, or a linseed oil-based long oil alkyd resin demonstrated high water repellency with CA between 100 and 130° and had minimal effect on surface gloss and did not change the color of coated wood [28]. Synthetic and natural epoxy resins and a rosin acid derivative as crosslinking agent have been used to prepare wood coatings which represented good adhesion to the wood, increased hardness and anti-fungal resistance which was ensured by limited water penetration into coated specimens [29]. However, the UV stability of such coatings was not tested.

**Figure 5:**
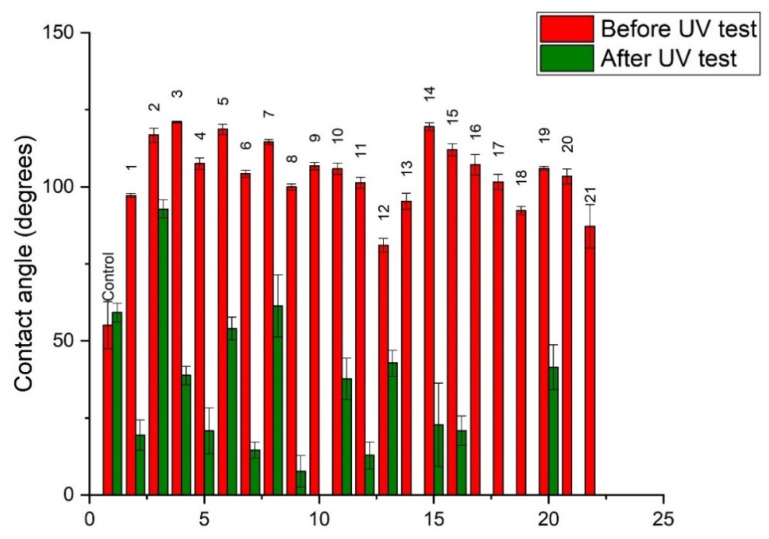
Water droplet surface contact angle (CA) after 30 s for untreated (Control) and M-PF resin coated birch wood before and after UV test.

To characterize the changes in surface hydrophobicity, CA of specimens treated with M-PF were determined before and after UV exposure. The CA values after UV exposure of the specimens decreased significantly and did not exceed 90° (Figure 5). Untreated wood showed no significant changes in CA values of surface after exposure to UV light. For specimens 9, 13, 16, 17, 18, 20, and 21, it was not possible to measure CA after exposure to UV light as water droplet instantly spilled over the surface of the wood. Only specimen 2 which was obtained using palmitoyl chloride (C_16_) and resin A with M_w_ = 266 g/mol showed hydrophobic effect after exposure to UV light as CA was ~90°. It seems that UV light caused splitting or destruction of long aliphatic chains in M-PF resin structure, and hydrophilic destruction products were formed on the surface of wood. However, this theoretical consideration needs to be confirmed in future tests, because also other researchers have not focused on CA changes after exposure to intensive UV light [25,26,28].

### 3.4. Surface Color after UV and Water Spray Test

**Figure 6:**
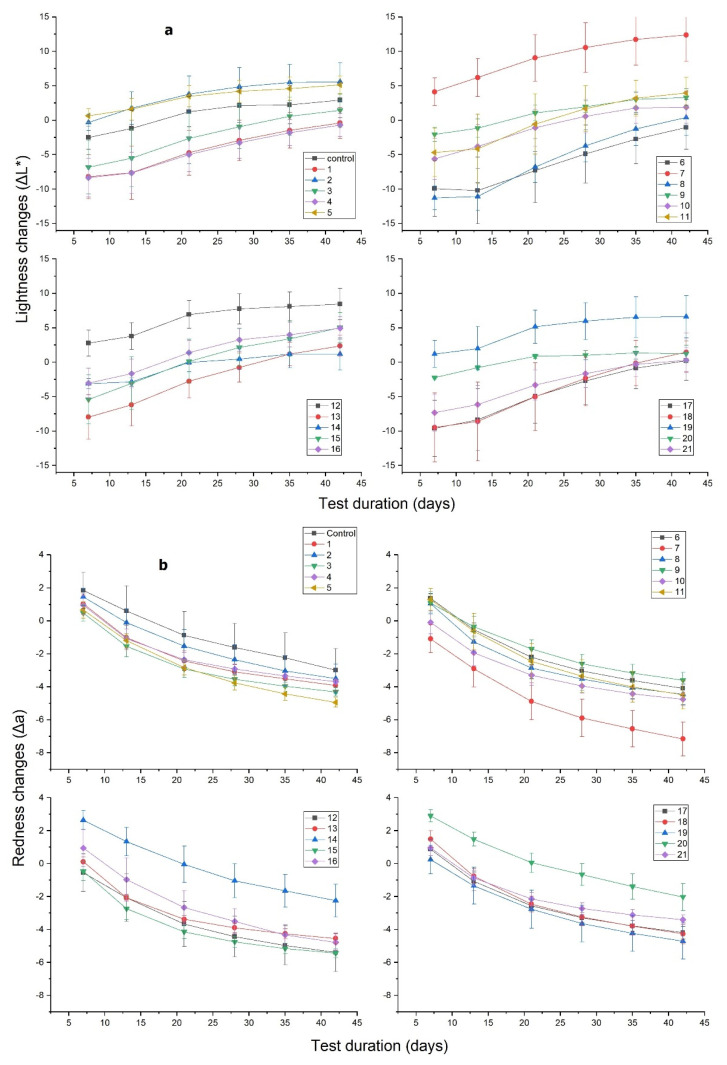

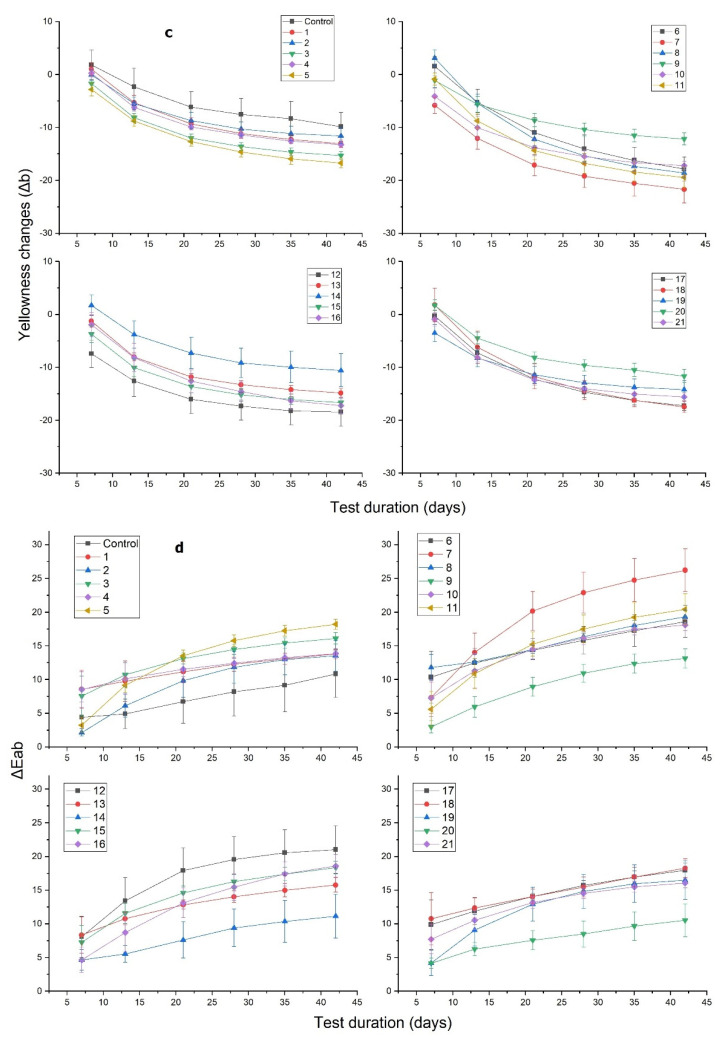
Changes in color of untreated (Control) birch wood and M-PF resin-coated wood after exposure to UV light and water spray during 1000 h in QUV camera: (**a**) Changes in the CIE parameter ΔL* (lightness); (**b**) changes in the CIE parameter Δa (red-green); (**c**) changes in the CIE parameter Δb (yellow-blue); (**d**) color difference parameter Δ*Eab*.

In order to simulate outdoor conditions, artificial weathering using a combination of UV radiation and moisture spraying was used. Moisture spraying recreated the effect of rain, mist, and dew. Results presented in Figure 6 show color parameters of M-PF resin-treated and untreated birch wood surface after UV weathering and water spraying, after a total test time of ~6 weeks (1000 h).

After 1000 h of UV weathering and water spraying, birch wood treated with M-PF resin as well as untreated wood became lighter (increasing ∆L*) compared to unweathered reference specimens. For M-PF resin-treated specimens, lightness increased by 5–10 units, but for untreated wood by ~5 units (Figure 6a). The redness parameter (Δa) decreased by 5 units for untreated wood and 4–6 units for all M-PF treated specimens (Figure 6b). Moreover, the yellowness parameter (Δb) changes were very similar and decreased by 12 units for untreated wood and 10–12 units for all M-PF treated specimens (Figure 6c).

The total color change (Figure 6d) for all M-PF treated wood specimens was between 10 and 27 units after 1000 h of UV light and water spray. Untreated birch wood Δ*Eab* was 11 units at the end of test and only specimens coated with M-PF resins 9, 14, and 20 showed similar total color changes. PF resins C, F, and H (M_w_ = 420, 292, and 549 g/mol) modified with palmitoyl chloride C_16_ and lauroyl chloride C_12_ (for specimen 9) were used to obtain these compounds. There was no clear correlation between used PF resins and their molecular weight or chain length of FAC to ensure better color stability in UV and water spray test.

Visual assessment of the specimens showed that the untreated birch wood turned gray with surface cracking after 1000 h of UV and water spraying. All the M-PF resin-treated specimens acquired similar gray color with surface cracks that differed considerably from the original color of the specimens prior to weathering. UV and water spray weathering test confirmed that all M-PF resins behaved similar in simulated conditions and were not stable against UV light and water spray. Thus, such M-PF resin use as surface treatment agent without additives is limited in real outdoor conditions.

Our previous studies showed that for plywood made of PF resin (M_w_ = 292, 528, 703, 884 g/mol), impregnated birch veneers after UV and water spray test acquired an uneven brownish color with some shades of grey that differed considerably from the original color of the specimens prior to weathering. As a result of UV light and water spraying, PF resin that was not fixed within the wood cell wall leached to the plywood surface, causing uneven discoloration patterns during simulated weathering. Thus, PF pre-polymer-impregnated birch veneers had better color stability [30].

## 4. Discussion

UV light caused splitting or destruction of long aliphatic chains in M-PF resin structure and hydrophilic destruction products were formed on the surface of wood. However, this theoretical consideration needs to be confirmed in future tests. M-PF resins could be used as additive in wood material coating formulations together with more stable UV compounds to ensure surface UV stability and hydrophobicity. In future, M-PF resins should be investigated in combination with different PF resins, although their structure is hydrophobic and emulsion would be difficult to obtain as there is water in PF resins. Probably, water should be removed and organic liquid should be added, and such mixture of M-PF and PF resins could be obtained and tested in terms of wood surface and bulk protection.

## 5. Conclusions

In the introduction we assumed that the surface hydrophobicity, weathering, and color stability of birch wood treated with M-PF resin oligomers can be improved. However, this theoretical consideration was confirmed only partly. The modification of PF resin hydroxymethyl groups with FAC significantly improved the resin performance in terms of surface hydrophobicity. It was possible to obtain transparent wood coatings which changed the natural birch wood color negligibly. The CA for most of the specimens was between 80 and 110° reaching up to 125° which indicated highly hydrophobic properties, but superhydrophobic surfaces with CA above 150° was not possible to obtain. It seems that using a longer chain FAC (C_16_–C_18_) compared to C_12_–C_14_ resulted in higher maximum surface hydrophobicity. Fewer M-PF resins showed improved color stability in UV light compared to untreated wood. Unfortunately, it seems that UV light caused splitting or destruction of long aliphatic chains in M-PF resin structure, and hydrophilic destruction products were formed on the surface of wood as CA after exposure to UV light decreased dramatically. For a few specimens, it was not possible to measure the CA as water droplet instantly spilled over the surface of the wood. UV and water spray weathering test confirmed that all M-PF resins behaved similar in simulated conditions and were not stable against UV light and water spray. Thus, such M-PF resin use as surface treatment agent without additives is limited in real outdoor conditions.

## Figures and Tables

**Table 1:** Characteristic parameters of PF resins used in the study.

Resin	Formaldehyde: Phenol: NaOH Molar Ratio	Synthesis Temperature/Duration (°C/h)	Viscosity (mPas)	Solid Content (%)	Free Formaldehyde (%)	pH	Mn (g/mol)	Mw (g/mol)
A	1.5-1-0.1	65/4	68	50.5	0.7	9.6	214	266
B	1.5-1-0.1	85/2	110	46.1	0.3	9.8	320	515
C	1.5-1-0.2	75/4	122	43.7	0.2	10.3	291	420
D	1.5-1-0.2	85/2	151	44.6	0.4	10.3	355	566
E	1.8-1-0.15	65/4	75	49.5	0.8	9.9	223	273
F	2-1-0.2	65/4	78	50.2	0.6	10.2	220	292
G	2-1-0.2	85/2	177	49.3	0.6	10.2	467	884
H	2-1-0.4	75/4	190	47.2	0.3	11.0	373	549
I	2-1-0.4	85/2	268	47.5	0.3	11.2	484	849

**Table 2:** Synthesis parameters of PF resin esterification reactions with FAC.

Modified PF Resin (M-PF)	PF Resin Used	Fatty Acid chloride (FAC)	Synthesis Temperature/Duration (°C/h)	FAC/PF Resin Ratio (mol/g)	Reaction Yield (M-PF/(PF Resin + FAC)) (%)
1	A	C_14_	60/2	0.0015	3
2	A	C_16_	60/2	0.0015	4
3	A	C_18_	60/2	0.0015	7
4	B	C_12_	60/2	0.0010	3
5	B	C_18_	50/2	0.0010	5
6	B	C_16_	50/3	0.0010	4
7	B	C_18_	60/2	0.0010	7
8	C	C_16_	60/2	0.0015	10
9	C	C_12_	60/3	0.0015	3
10	D	C_18_	50/2	0.0010	10
11	D	C_16_	60/2	0.0015	10
12	E	C_18_	60/2	0.0010	9
13	F	C_12_	60/2	0.0015	4
14	F	C_16_	60/2	0.0015	5
15	F	C_18_	60/2	0.0015	6
16	G	C_16_	60/2	0.0015	16
17	G	C_14_	60/2	0.0015	6
18	G	C_18_	60/2	0.0010	17
19	G	C_16_	60/3	0.0010	11
20	H	C_16_	50/3	0.0010	26
21	I	C_14_	50/3	0.0015	23

## References

[1] Xie Y., Krause A., Militz H., Mai C. (2006). Coating Performance of Finishes on Wood Modified with an N-Methylol Compound. Prog. Org. Coat..

[2] Schmid E.V. (1988). Exterior Wood-Coatings and the Glass-Transition Temperature. Polym. Paint Colour J..

[3] Evans P., Vollmer S., Kim J., Chan G., Kraushaar Gibson S. (2016). Improving the Performance of Clear Coatings on Wood through the Aggregation of Marginal Gains. Coatings.

[4] Altgen M., Militz H. (2017). Thermally Modified Scots Pine and Norway Spruce Wood as Substrate for Coating Systems. J. Coat. Technol. Res..

[5] Williams R.S. (1983). Effect of Grafted UV Stabilizers on Wood Surface Erosion and Clear Coating Performance. J. Appl. Polym. Sci..

[6] Richter H.G., Schwab E., von Arps-Aubert T., Nock H.P. (2004). Quality Assessment of Laminated Window Scantlings from Mixed Tropical Hardwoods after Long-Term Exposure to Weathering. J. Trop. For. Sci..

[7] Rowell R., Bongers F. (2015). Coating Acetylated Wood. Coatings.

[8] Evans P.D., Wallis A.F.A., Owen N.L. (2000). Weathering of Chemically Modified Wood Surfaces. Wood Sci. Technol..

[9] Xiao Z., Xie Y., Adamopoulos S., Mai C. (2012). Effects of Chemical Modification with Glutaraldehyde on the Weathering Performance of Scots Pine Sapwood. Wood Sci. Technol..

[10] Xie Y., Krause A., Militz H., Turkulin H., Richter K., Mai C. (2007). Effect of Treatments with 1,3-Dimethylol-4,5-Dihydroxy-Ethyleneurea (DMDHEU) on the Tensile Properties of Wood. Holzforschung.

[11] Feist W.C., Sell J. (1987). Weathering Behavior of Dimensionally Stabilized Wood Treated by Heating under Pressure of Nitrogen Gas. Wood Fiber Sci..

[12] Altgen M., Adamopoulos S., Militz H. (2015). Wood Defects during Industrial-Scale Production of Thermally Modified Norway Spruce and Scots Pine. Wood Mater. Sci. Eng..

[13] Mantanis G., Lykidis C. (2015). Evaluation of Weathering of Furfurylated Wood Decks after a 3-Year Outdoor Exposure in Greece. Drvna Ind..

[14] Kielmann B.C., Mai C. (2016). Application and Artificial Weathering Performance of Translucent Coatings on Resin-Treated and Dye-Stained Beech-Wood. Prog. Org. Coat..

[15] Jämsä S., Ahola P., Viitaniemi P. (2000). Long-term Natural Weathering of Coated ThermoWood. Pigment. Resin Technol..

[16] Beckers E.P.J., Meijer M., Militz H., Stevens M. (1998). Performance of Finishes on Wood That Is Chemically Modified by Acetylation. J. Coat. Technol..

[17] Tarkow H., Southerland C.F., Seborg R.M. (1966). Surface Characteristics of Wood as They Affect Durability of Finishes Part 1. Surface Stabilizazion.

[18] Black J.M., Laughnan D.F., Mraz E.A. (1979). Forest Products Laboratory Natural Finish.

[19] Lu Y., Xiao S., Gao R., Li J., Sun Q. (2014). Improved Weathering Performance and Wettability of Wood Protected by CeO2 Coating Deposited onto the Surface. Holzforschung.

[20] Wang K., Dong Y., Yan Y., Zhang W., Qi C., Han C., Li J., Zhang S. (2017). Highly Hydrophobic and Self-Claning Bulkwood Prepared by Grafting Long-Chain Alkyl onto Wood Cell Walls. Wood Sci. Technol..

[21] Wimmer R., Kläusler O., Niemz P. (2013). Water Sorption Mechanisms of Commercial Wood Adhesive Films. Wood Sci. Technol..

[22] (2019). DIN EN ISO 3251:2019 Paints, Varnishes and Plastics; Determination of Non-Volatile-Matter Content.

[23] (1997). DIN EN ISO 9397: 1997 Plastics—Phenolic Resins—Determination of Free-Formaldehyde Content—Hydroxylamine Hydrochloride Method.

[24] Grinins J., Biziks V., Rizikovs J., Irbe I., Militz H. (2021). Evaluation of Water Related Properties of Birch Wood Products Modified with Different Molecular Weight Phenol-Formaldehyde Oligomers. Holzforschung.

[25] Liu F., Gao Z., Zang D., Wang C., Li J. (2015). Mechanical Stability of Superhydrophobic Epoxy/Silica Coating for Better Water Resistance of Wood. Holzforschung.

[26] Janesch J., Arminger B., Gindl-Altmutter W., Hansmann C. (2020). Superhydrophobic Coatings on Wood Made of Plant Oil and Natural Wax. Prog. Org. Coat..

[27] Rajput S.D., Mahulikar P.P., Gite V.V. (2014). Biobased Dimer Fatty Acid Containing Two Pack Polyurethane for Wood Finished Coatings. Prog. Org. Coat..

[28] Janesch J., Gusenbauer C., Mautner A., Gindl-Altmutter W., Hansmann C. (2021). Efficient Wood Hydrophobization Exploiting Natural Roughness Using Minimum Amounts of Surfactant-Free Plant Oil Emulsions. ACS Omega.

[29] Rosu L., Mustata F., Rosu D., Varganici C.-D., Rosca I., Rusu T. (2021). Bio-Based Coatings from Epoxy Resins Crosslinked with a Rosin Acid Derivative for Wood Thermal and Anti–Fungal Protection. Prog. Org. Coat..

[30] Grinins J., Biziks V., Marais B.N., Rizikovs J., Militz H. (2021). Weathering Stability and Durability of Birch Plywood Modified with Different Molecular Weight Phenol-Formaldehyde Oligomers. Polymers.

